# Supplementary material for: Metabolite-Mediated Responses of Phyllosphere Microbiota to Rust Infection in Two Malus Species
Source: Microbiol Spectr. 2023 Mar 14;11(2):e03831-22. doi: 10.1128/spectrum.03831-22 (PMC10101083; doi:10.1128/spectrum.03831-22)
Supplement: Supplemental file 10 — Figures S1 to S4. Download spectrum.03831-22-s0010.pdf, PDF file, 0.5 MB [file spectrum.03831-22-s0010.pdf]

Supplemental Figure 1

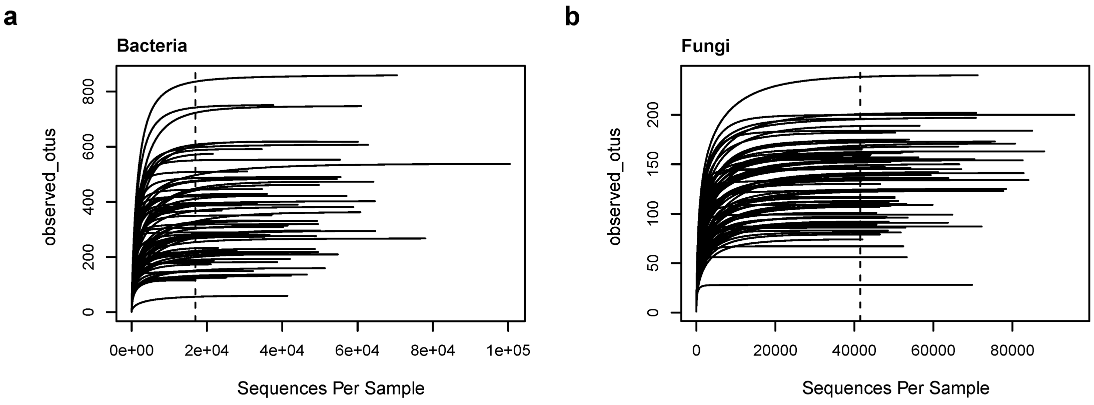

Fig. S1 Rarefaction curves of bacterial (**a**) and fungal (**b**) amplicon sequence variants (ASV) for each sample.

Supplemental Figure 2

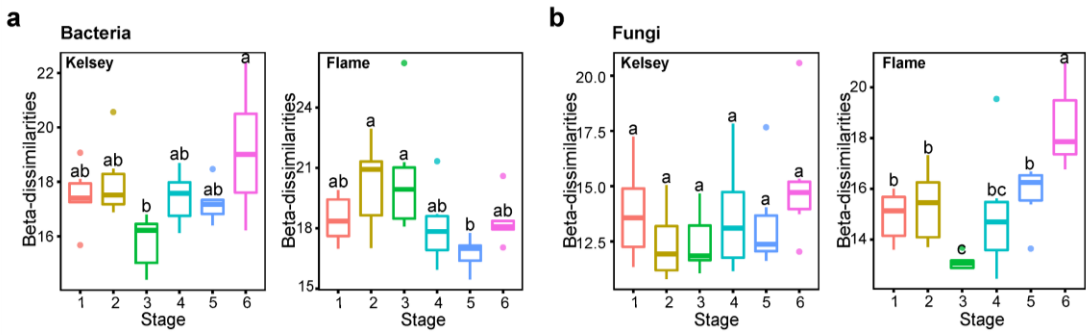

Fig. S2 Beta-dissimilarities based on weighted unifrac distances of bacterial (**a**) and fungal (**b**) communities in the two *Malus* cultivars (*M*. 'Flame' and *M*. 'Kelsey').

Supplemental Figure 3

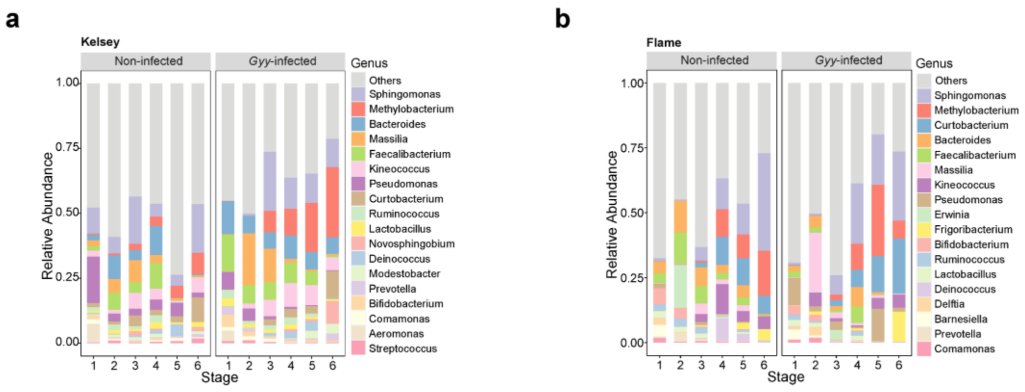

Fig. S3 Taxonomic compositions of the phyllosphere bacterial community at the genus level in the two *Malus* cultivars (*Malus* ‘Flame’ and *M*. ‘Kelsey’).

Supplemental Figure 4

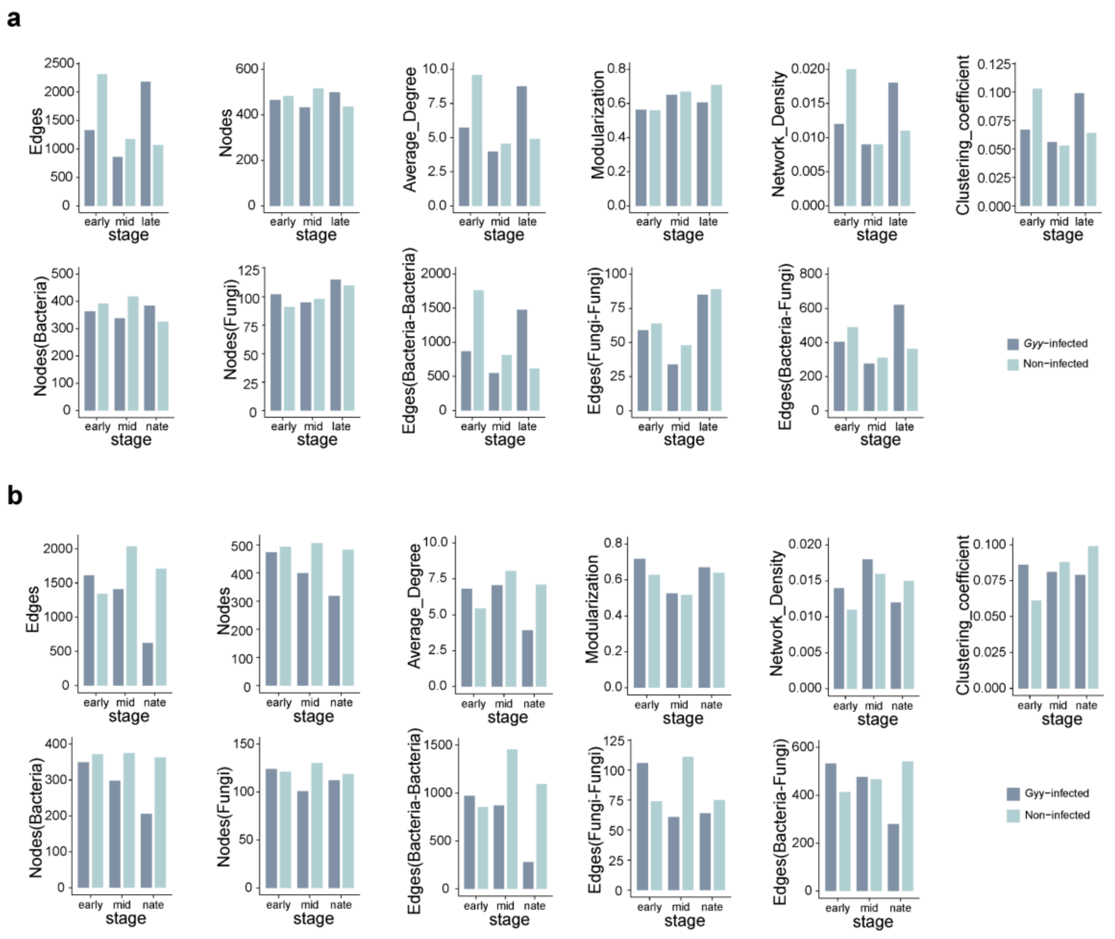

Fig. S4 Changes in patterns of phyllosphere microbial network association indices of non-infected and *Gymnosporangium yamadae* (*Gyy*)-infected leaves for *Malus* 'Kelsey' (**a**) and *M*. 'Flame' (**b**) at different stages (i.e., early stages, mid stages and late stages).
